# Does treatment with clomipramine reduce cat psychogenic alopecia?

**DOI:** 10.18849/ve.v7i2.573

**Published:** 2022-06-29

**Authors:** Anne-Claude Griesser+

**Keywords:** CATS, CLOMIPRAMINE, COMPULSIVE DISORDER, OVERGROOMING, PYSCHOGENIC ALOPECIA, STRESS RELATED BEHAVIOUR, TRICYCLIC ANTIDEPRESSANTS

## Abstract

PICO question

In cats with psychogenic alopecia, is overgrooming reduced by the use of clomipramine compared to untreated cats?

Clinical bottom line

Category of research question

Treatment

The number and type of study designs reviewed

One pseudo-randomised controlled study

Strength of evidence

Weak

Outcomes reported

Effect of clomipramine using owner report of number, intensity, and / or duration of grooming episodes, owner reported clinical improvement, and veterinary measured alopecia, extent of tissue damage, and hair regrowth

Conclusion

The only controlled study found no evidence that clomipramine alone is effective in reducing grooming episodes, alopecia, or improved hair regrowth. Further research with randomised, double blind controlled trials and limitation of confounding factors is required to determine the efficacy of clomipramine alone or in addition to behavioural / environmental therapies

How to apply this evidence in practice

The application of evidence into practice should take into account multiple factors, not limited to: individual clinical expertise, patient’s circumstances and owners’ values, country, location or clinic where you work, the individual case in front of you, the availability of therapies and resources.

Knowledge Summaries are a resource to help reinforce or inform decision making. They do not override the responsibility or judgement of the practitioner to do what is best for the animal in their care.

## Clinical scenario

A 6 year old female neutered cat exhibits recurrent episodes of hair pulling and overgrooming without underlying medical cause. A full skin work up has been carried out to exclude fleas or flea allergy. Despite environmental changes reducing some stressors the cat continues overgrooming. The cat’s abdominal and inner thigh baldness worries the owner who would like to know if pharmacotherapy might prevent her cat’s overgrooming. The veterinarian knows that Clomicalm^®^ by Novartis AG has good efficacy and is well tolerated in case of feline urine spraying and wonders therefore if this medication could also be efficacious to treat psychogenic alopecia.

## The evidence

Based on the only controlled study comparing clomipramine efficacy with placebo, there is no evidence that clomipramine alone reduces psychogenic alopecia in cats. Controlled trial evidence was weakened by small patient numbers and confounded with environmental changes.

## Summary of the evidence

**Mertens et al. (2006):** 

Population:	Cats of any breed, sex, or age with an history of non-inflammatory alopecia, referred to a behaviour or dermatology consultation of the College of Veterinary Medicine at the University of Minnesota. Cats should not be sensitive to tricyclic antidepressants (TCAs), or having received selective serotonin reuptake inhibitors (SSRIs) or TCAs or monoamine oxidase inhibitors (MAOIs) within the previous 4 weeks, or glucocorticoids within the previous 8 weeks. Cats with skin biopsy showing sign of inflammation were tested for food or environmental allergies and were excluded if positive.
Sample size:	25 cats were included in the study after a skin analysis was performed to confirm self-inflicted alopecia without any dermatologic condition. Since data were not completely recorded or treatment not administered properly, two cats were withdrawn from the study. In addition, one cat exhibited urinary obstruction at day 18 resulting in withdrawal from the study. Therefore, 22 cats completed the study.
Intervention details:	Treatment groups Cats were assigned alternatively to either a group receiving placebo (n = 13) or a group receiving clomipramine hydrochloride (0.5 mg/kg PO *q* 24 hours) (n = 12). Placebo and clomipramine were in similar gel capsules. Investigators and owners were blind to cat treatment condition (i.e., clomipramine vs placebo). Neither formal behavioural nor environmental modification plans were implemented in either group. Owners were however requested to prevent reinforcement of overgrooming and to stop positive punishment. Experimental timeline Treatment Either placebo or clomipramine was administered from day 1 to day 56. Follow-up phase from day 57 until day 84. Specific role of owners 7 days before starting treatment (day 1–7), owners completed a background questionnaire and performed a baseline assessment. The latter was recorded in a logbook. It encompassed: Total number of episodes of licking, chewing, and hair pulling / 24 hours. Specific behaviours: anxiousness, calmness, use of cat litter, interactions with human and household pets. Special events and environmental changes. This assessment was repeated daily from day 1 until day 84. Owners were also asked to record the time of treatment and the occurrence of any adverse events. On day 84, owners were asked to qualitatively assess the overall changes exhibited by their cat from day 1 to day 84. Specific role of the professional team On day 0 (i.e., 1 day before the start of the treatment), day 28, day 56 and day 84: Laboratory tests were performed. A dermatologist measured the extent of the alopecia and the degree of hair regrowth. An outline of the dorsum, ventrum and lateral sides of the body were drawn on a graph paper to record the area of alopecia and hair regrowth at each consultation. A behaviourist made a behavioural assessment (no detail is provided on this assessment). On day 0 and day 28, ECG were performed. On day 28 and day 56, the team measured owner’s compliance with treatment administration by checking the logbook, the number of remaining capsules and volume of liquid.
Study design:	Double-blind placebo-controlled trial.
Outcome studied:	Grooming episodes: Daily episodes of licking, chewing, and hair pulling from day 0 to day 84, based on a specific protocol (similar time and duration of daily observations by same observer i.e., owner). Alopecia scores from 1–5, on day 28, day 56 and day 84, in comparison to day 0: Score 1 reduced by >75% Score 2 reduced by >50–75% Score 3 reduced by >25–50% Score 4 reduced by < 25% Score 5 new areas of alopecia or no change from day 0 Mean hair regrowth scores: Hair length adjacent to the alopecia area was measured on day 0 and day 28, day 56 and day 84 and the percent reduction between the adjacent area to alopecia and normal hair length was measured. A range score from 1–5 was used to describe these differences in hair regrowth. Score 1 reduced by >75% Score 2 reduced by >50–75% Score 3 reduced by >25–50% Score 4 reduced by <25% Score 5 no change from Day 0 4. **Adverse events**: qualitative owners’ observation, clinical signs, ECG, complete blood counts, thyroxine (T4) and serum biochemicals. 5. **General evaluation by the owners.**
Main Findings (relevant to PICO question)	Grooming episodes No significant differences in the mean number of grooming episodes between clomipramine and placebo groups (analysis of variance; P = 0.13). Alopecia scores No differences in the mean scores between the two treatment groups (Kruskal-Wallis test) at day 28 (P = 0.44), day 56 (P = 0.06) and post-treatment at day 84 (P = 0.39). Hair regrowth No differences were found in mean scores between the two treatment groups (Kruskal-Wallis test) at day 28 (P = 0.18), day 56 (P = 0.48) and post-treatment at day 84 (P = 0.34). Adverse events Clomipramine group: No abnormalities were attributed to clomipramine on the ECG or laboratory tests Urethral obstruction after 18 days of treatment in one cat Lethargy in five cats Constipation in one cat Reduced appetite in one cat Reduced interaction with owner in four cases Difficulty in administering medication in two Placebo group: Intermittent vomiting in two cats Increased search for owner attention by one cat during the first 2 weeks of the study. General evaluation by owners: 7/11 (64%) owners in the clomipramine group reported behaviour and hair coat improvement of at least 50%. 3/11 (27%) owners in the placebo group reported an improvement of at least 50%.
Limitations:	Study design: With 25 enrolled cats, the sample size was undersized, and no power calculation was reported. The statistical treatment difference to be detected to conclude that clomipramine was superior to placebo was not specified. No randomisation but rather alternating allocation of the enrolled cats to clomipramine or placebo group, leading to a possible bias in the selection of cases and non-comparability of the two treatment groups. Since the paper does not include a table of the cats details for each group, it is not possible for the reader to appraise if the two groups were comparable or if there was any bias in the grouping. While the term ‘investigators’ may include the three authors of the paper only, it is not clear who was specifically blinded to the type of treatment, thus preventing the assessment of possible biases. Lack of justification for the recommendations to owners of stopping negative punishment or prevention of reinforcement for grooming in addition to a lack of report on the number of involved cats, prevent the reader from evaluating the impact of this environmental modification on the outcome. No comprehensive measurement of the severity of alopecia including problem duration which might impact outcome. No justification of alopecia reduction and hair regrowth as outcome and whether these two measures were sensitive and specific enough since hair regrowth may differ from one cat to another. No measurement of cat stress with either a validated instrument, or with blood or salivary cortisol level, or both. The authors reported that some enrolled cats may have had dermatologic issues such as demodicosis or cheyletioellosis. No parasiticide response trial was performed for all the cats before inclusion in the study. Furthermore, it was possible that some of the enrolled cats had food allergies and/or atopic dermatitis while the histopathological evidence of inflammation in non-affected skin failed to demonstrate food allergies in some cases. Lack of details whether postprandial vomiting or spitting out was recorded, as part of measuring owner compliance with clomipramine administration. Clomipramine is known to have a bitter taste. Results: Lack of information for determining if clomipramine and placebo groups were homogeneous or if some cats’ characteristics were overrepresented in one group or in the other. Over representation of exclusively indoor cats and multi-cat households (88%) compared to the general population of domestic cats leading to a potential limit for generalising to the overall household cat population. Baseline assessment and results were presented on a graph without explanation nor discussion of 30% higher average number of grooming episodes in the clomipramine versus placebo group at the baseline assessment making difficult for the readers to appraise this difference between both groups. Lack of precise mean values and standard deviations of the average number of grooming episodes, alopecia score, and hair regrowth score, prevents the reader from evaluating the variance of results. No information about how alopecia size was factored into interpretation of results. Lack of detail about the exhibited grooming behaviours and how they were observed and reported. Conflict of interest: None declared

Appraisal, application and reflection

One pseudo-randomised double-blind, placebo-controlled trial study that fully addressed the PICO question was reviewed.

The controlled study (Mertens et al., 2006) revealed no significant difference between clomipramine and placebo groups, either in the number of grooming episodes throughout the clomipramine treatment period and after its discontinuation or in the score of alopecia and hair regrowth after 28 and 56 days medication and 28 days after stopping the medication.

With 25 enrolled patients in the trial by Mertens et al. (2006), the sample size was too small to test whether clomipramine is superior to placebo in reducing overgrooming. Furthermore, the variability in the pharmacokinetics of clomipramine in cats (Lainesse et al., 2007) may also have reduced the ability to detect statistical significance. In addition, the 25 patients were not randomly assigned to the two treatment groups, with a possible selection bias and may have limited the comparison. Even if Mertens et al. (2006) did not report any differences across the two groups, it is indeed noticeable that the average number of grooming episodes was 30% higher in the clomipramine group (45) than in the placebo group (30) at the baseline assessment.

Mean grooming episodes decreased by 57% after 56 days of treatment and a rebound after discontinuation of treatment was observed in the clomipramine group, whereas the dynamics in the placebo group did not show the same pattern. It is therefore crucial to ask whether the inclusion of the entire observation period in the calculation of therapeutic efficacy on grooming episodes was relevant since evidence indicates a possible rebound of symptoms when antidepressants are discontinued (Henssler et al., 2019). Therefore, two separate assessments of the efficacy (1) during the treatment period and (2) its duration after cessation of treatment may have been more appropriate. While the owners were blind to treatment conditions, their ratings may have had unexpected intra- and inter-observer variability effects. That said, twice as many owners (7/11) in the clomipramine group found their cat's behaviour and coat improved compared to the placebo group (3/11).

Since there was no information on how the scoring system was validated, it is debatable whether alopecia and hair regrowth scores were specific enough or sufficient to measure medication efficacy while hair growth can depend on different factors such as season, sex, breed and nutrition (Affolter & Moore, 1994; and Hendriks et al., 1997). In addition, there was no indication for psychogenic alopecia severity such as duration of overgrooming. Neither the context of its exhibition nor the size of the area affected was discussed by the authors as a confounding factor.

Cat psychogenic alopecia is considered a behavioural disorder that may be precipitated by emotional stress triggered by the environment (Virga, 2003). Therefore, stopping positive punishment may have alleviated the stress-overgrooming cycle, thus limiting the interpretation of results of both groups while it is not reported how positive punishment by owners was distributed across the clomipramine group and the placebo group. It should be noted that one before and after study (Seksel & Lindeman, 1998), two case series (Overall & Dunham, 2002; and Sawyer et al., 1999) and one case study (Talamonti et al., 2017) reported that implementation of a behavioural and environmental modification plan along with clomipramine provided a sustained reduction in overgrooming among cats with psychogenic alopecia. Although subjective assessment by owners may have overestimated the positive effects of the treatment combination, these non-controlled study findings suggest Mertens et al. (2006) cannot be interpreted without consideration of the environmental changes that may have occurred for some cats enrolled in the study.

In Mertens et al. (2006), cats were recruited from second-line consultations which constitutes a selection bias. This should be considered but does not constitute an obvious limit to generalisation. Psychogenic alopecia is a complex condition that can be challenging to diagnose and treat in a first-line consultation. Furthermore, it is good practice that such cases are referred to specialists.

Multi-cat households (22/25) or indoor cats (23/25) were overrepresented in the sample of the study by Mertens et al. (2006), which could limit the generalisation of the results. However, it is known that risk factors for developing a behavioural problem in cats, such as overgrooming include living with other cats or animals (Luescher, 2003), or not having access to the outdoors (Virga, 2003).

The reviewed study (Mertens et al., 2006) found that clomipramine alone was not effective in treating psychogenic alopecia in cats. This study was of limited quality and had several confounding factors that may explain the results. Therefore, further research with randomised, double-blind controlled trials and limitation of confounding factors is needed to substantiate the efficacy of clomipramine alone or in addition to behavioural / environmental therapies. It should be indeed noted that various non-controlled studies (Overall & Dunham, 2002; Sawyer et al., 1999; Seksel & Lindeman, 1998; and Talamonti et al., 2017) suggested that if a behavioural and environmental modification plan was set up alongside the clomipramine treatment in cats with psychogenic alopecia the exhibition of overgrooming might diminish or even discontinue.

## Methodology Section

**Search Strategy:** 

Databases searched and dates covered:	CAB Abstracts on OVID Platform (1973–2021) PubMed via NCBI Website (1900–2021) Web of Science on OVID Platform (1970–2021)
Search strategy:	CAB Abstracts: (cats or cat).mp. [mp=abstract, title, original title, broad terms, heading words, identifiers, cabicodes] (felis catus or feline* or felid*).mp. [mp=abstract, title, original title, broad terms, heading words, identifiers, cabicodes] (psychogenic alopecia or over-grooming or Hair loss or licking).mp. [mp=abstract, title, original title, broad terms, heading words, identifiers, cabicodes] (obsessive-compulsive disorder or compuls*adj$ behavio*).mp. [mp=abstract, title, original title, broad terms, heading words, identifiers, cabicodes] (abnormal behaviour or behavior).mp. [mp=abstract, title, original title, broad terms, heading words, identifiers, cabicodes] behaviour disorders/ (skin diseases or pruritus).sh. (clomipramine or clomicalm or anafranil).mp. [mp=abstract, title, original title, broad terms, heading words, identifiers, cabicodes] (1 or 2) and (3 or 7 or 4 or 5) and 8 **PubMed:** ("cats"[MeSH Terms] OR ("cat"[All Fields] OR ("cats"[MeSH Terms] OR "cats"[All Fields]))) AND ("obsessive compulsive disorder"[MeSH Terms] OR "over-grooming"[All Fields] OR "grooming"[MeSH Terms] OR ("groomed"[All Fields] OR "grooming"[MeSH Terms] OR "grooming"[All Fields] OR "groom"[All Fields] OR "groomings"[All Fields] OR "grooms"[All Fields]) OR "groom*"[All Fields] OR "alopecia"[MeSH Terms] OR (("psychogenic"[All Fields] OR "psychogenically"[All Fields] OR "psychogenicity"[All Fields] OR "psychogenous"[All Fields]) AND ("alopecia"[MeSH Terms] OR "alopecia"[All Fields] OR "alopecias"[All Fields])) OR ("alopecia"[MeSH Terms] OR "alopecia"[All Fields] OR ("hair"[All Fields] AND "loss"[All Fields]) OR "hair loss"[All Fields]) OR "itch*"[All Fields] OR "pruritus"[MeSH Terms] OR "dermatitis/veterinary"[MeSH Terms] OR "bald*"[All Fields] OR "stereot*"[All Fields] OR "anxiety"[MeSH Terms]) AND ("clomipramine"[MeSH Terms] OR ("clomipramine"[MeSH Terms] OR "clomipramine"[All Fields] OR "clomipramine s"[All Fields]) OR ("clomicalm"[All Fields] OR "clomipramine"[MeSH Terms] OR "clomipramine"[All Fields] OR "clomipramine s"[All Fields]) OR ("clomipramine"[MeSH Terms] OR "clomipramine"[All Fields] OR "anafranil"[All Fields])) Web of Science: ((ALL=(cats OR cats OR feline* OR felis OR felid*)) AND ALL=(clomipramine OR clomicalm OR anafranil )) AND ALL=(compulsive behaviour OR compulsive behavior OR over-grooming OR grooming OR hair-loss OR itching OR stereot* or bald* OR licking OR alopecia OR dermatitis OR stereot* OR behaviour disorder OR behavior disorder)
Dates searches performed:	11 Dec 2021

**Exclusion / Inclusion Criteria:** 

Exclusion:	Pre-defined exclusion criteria: non-English language, proceedings, book chapter, popular press.
Inclusion:	Any comparative (control group utilised) study published in a peer-reviewed journal in which the effect of clomipramine on psychogenic alopecia or overgrooming in cats was studied.

**Search Outcome:** 

Database	Number of results	Excluded – Not relevant to PICO question	Excluded – Not English language	Excluded – Chapter of book or proceedings	Excluded – Duplicate	Total relevant papers
CAB Abstracts	32	18	3	6	5	0
PubMed	8	6	1	0	0	1
Web of Science	37	23	6	1	7	0
Total relevant papers when duplicates removed						1

## Conflict of interest

The authors declare no conflicts of interest.
